# Supplementary material for: Is a meditation retreat the better vacation? effect of retreats and vacations on fatigue, emotional well-being, and acting with awareness
Source: PLoS One. 2021 Feb 8;16(2):e0246038. doi: 10.1371/journal.pone.0246038 (PMC7869997; doi:10.1371/journal.pone.0246038)
Supplement: S1 File — (PDF) [file pone.0246038.s001.pdf]

S1 File: Recruitment emails

Email-invitation to individuals planning to participate on a retreat

Dear Summer Dathun participants,

This summer, our Karme Choling dathun program has the opportunity to participate in an international research project studying the meditation retreat experience. This study will be the first to assess the effects of the retreat experience in comparison to leisure holiday vacations on several psychological scales that are related to mindfulness, relaxation and general health. Other Shambhala residential land centers, including the Shambhala Mountain Center, Dechen Choling, and other Shambhala meditation retreats in Europe will also be participating in this online survey. Data collected from the surveys will be used to study the effects of meditation retreats, and will be used to develop a new assessment tool to better understand and study perceptions of self-worth-- the Basic Goodness Questionnaire -- which will be the first of its kind.

Participation in the study is voluntary and anonymous. In a few days, you will receive an email inviting you to participate in the Meditation Retreat Study, with a link connecting you to the online questionnaire. You will be asked a series of questions regarding your experiences, thoughts, and feelings before and after your meditation retreat. The online survey should take about 10-15 minutes to complete. You will receive an email with the online survey three times: 10-days before your retreat, and 10-days and 66-days after you return from your retreat.

We hope that you will participate in this study, and thank you for taking the time to answer the questionnaire. It is our hope that Shambhala International will be able to join the growing body of mindfulness research and believe that the important work that we do here at Shambhala retreat centers may benefit from the results of these studies.

The study is affiliated with the Medical University of Vienna, Austria, and will be conducted by Dr. Otto Pichlhoefer, MD (shastri in the Vienna Shambhala sangha), Dr. Gerhard Blasche, PhD (Medical University of Vienna), and Adrienne Chang (PhD candidate, Miami University, USA). For further information, please send an email to MeditationRetreatStudy@gmail.com.

Thank you kindly for your participation!

Email-invitation to individuals to participate in the retreat-study as control-group if planning a holiday vacation

Dear Shambhala sangha member,

This summer, the Shambhala residential land centers in the USA and Europe will be participating in an international research project studying the meditation retreat experience. The experience of the retreat participants will be compared to a control group of people who go on a leisure vacation of similar duration.

We need your participation too!

- If you are going on a holiday vacation in the upcoming months, we invite you to participate in our 10-minute online questionnaire. Please reply to this message (or email to

MeditationRetreatStudy@gmail.com) and tell us the start and end dates of your planned vacation, and we will send you the link to the online questionnaire at the appropriate time.

- If you already have gone on vacation this year, please also respond stating “I have already gone on vacation”.

- If you do not plan to go on vacation this year at all, please also reply stating this fact.

The Meditation Retreat Study will be the first to assess the effects of the meditation retreat experience in comparison to leisure holiday vacations on several psychological scales that are related to mindfulness, relaxation and general health.

The short online survey will ask you questions about your thoughts, feelings, and experiences before and after your vacation. Participation in the online study is voluntary and all data collected from the online survey will be anonymous.

The study is affiliated with the Medical University of Vienna, Austria, and will be conducted by Dr. Otto Pichlhoefer, MD (Shastri in the Vienna Shambhala sangha), Dr. Gerhard Blasche, PhD (Medical University of Vienna), and Adrienne Chang (PhD candidate, Miami , USA).

We are very excited by the prospect of involving Shambhala meditation retreat centers into the rapidly growing body of mindfulness research, and believe that the outcomes from this study will provide valuable data and information on the meditation retreat experience, and the important work being done by the Shambhala retreat centers.
