# Supplementary material for: Is a meditation retreat the better vacation? effect of retreats and vacations on fatigue, emotional well-being, and acting with awareness
Source: PLoS One. 2021 Feb 8;16(2):e0246038. doi: 10.1371/journal.pone.0246038 (PMC7869997; doi:10.1371/journal.pone.0246038)
Supplement: Attachment — Submitted filename: Letter to authors_Plos One.docx [file pone.0246038.s003.docx]

This manuscript aims to address timely research questions on the effects of intensive meditation practice, answers to which are usually limited by an absence of meaningful control groups. A clear merit is the novelty and elegance of the authors’ methodological approach. This study has, in principle, the potential to offer an important contribution to the ongoing debate on the efficacy of intensive meditation practice. However, there are some important conceptual points that need to be addressed before this manuscript could be considered for publication. Please regard the suggestions below as an attempt to improve the positive impact of this study on this nascent research field.

Major point:

Overall, the description and discussion of meditation practice is too general and broad. A more careful presentation of the complexities and nuances of contemplative research is needed, including a discussion of theoretical frameworks of meditation practices through which the type of meditation practiced on these retreats can be conceptualised. Specific suggestions are offered below.

Introduction

1. The terms “mindfulness meditation” and “meditation” are often used interchangeably. In the introduction, please offer a more nuanced definition of meditation as a family of diverse practices that include but are not limited to mindfulness meditation. Please refer to landmark papers including:

Dahl CJ, Lutz A, Davidson RJ. Reconstructing and deconstructing the self: cognitive mechanisms in meditation practice. Trends in cognitive sciences. 2015 Sep 1;19(9):515-23.

Lutz A, Slagter HA, Dunne JD, Davidson RJ. Attention regulation and monitoring in meditation. Trends in cognitive sciences. 2008 Apr 1;12(4):163-9.

Van Dam NT, Van Vugt MK, Vago DR, Schmalzl L, Saron CD, Olendzki A, Meissner T, Lazar SW, Kerr CE, Gorchov J, Fox KC. Mind the hype: A critical evaluation and prescriptive agenda for research on mindfulness and meditation. Perspectives on psychological science. 2018 Jan;13(1):36-61.

2. It is unclear why the manuscript starts off with a general statement about books published on “mindfulness”. Please consider deleting the first sentence of the introduction.

3. It is not clear why an elaborate presentation of research on mindfulness-based interventions is needed in the context of the present manuscript. On several levels, intensive meditation retreats differ markedly from standard mindfulness-based programmes. Please consider embedding the narrative of this manuscript within previous research on meditation retreats.

4. Please add a discussion of the systematic reviews and theoretical papers listed below. Please also use them to elaborate on the limitations and future directions of research on meditation retreats.

McClintock AS, Rodriguez MA, Zerubavel N. The effects of mindfulness retreats on the psychological health of non-clinical adults: A meta-analysis. Mindfulness. 2019 Aug 15;10(8):1443-54.

Khoury B, Knäuper B, Schlosser M, Carrière K, Chiesa A. Effectiveness of traditional meditation retreats: A systematic review and meta-analysis. Journal of Psychosomatic Research. 2017 Jan 1;92:16-25.

King BG, Conklin QA, Zanesco AP, Saron CD. Residential meditation retreats: their role in contemplative practice and significance for psychological research. Current opinion in psychology. 2019 Aug 1;28:238-44.

5. “Propelled by research and relatively distanced from its spiritual roots, mindfulness radiated through medicine into society at large.” Please consider reframing this. It seems that the retreats included in this study took place within an explicitly Buddhist framework.

6. Page 5, line 113: “While a meditation retreat aims at developing non-judgmental present moment awareness through meditation practice, …”. This is too broad and general. What types of meditation were primarily practised during these particular retreats (see comment 4 below)? Please describe why you have chosen retreats that have focussed primarily on “mindful-awareness”. Also, the term “mindful-awareness” is not commonly used in meditation research. Please clarify and specify the terminology in accordance with published conceptual frameworks (e.g. Dahl et al. 2015).

Methods

7. Page 7, line 213: “The formal sitting practice of meditation taught at these retreats is mindful-awareness, where the practitioner learns to focus their attention on an object of meditation, often the breath, to cultivate awareness of one’s inner world: thoughts, emotions, sensations, perceptions.” Please elaborate on the type of practice that was used, its perceived goals, and the cognitive mechanism it primarily cultivates. Please try to embed this type of practice within a theoretical taxonomy of meditation practices (e.g. Dahl et al. 2015) to differentiate it from other practices.

Reference: Dahl CJ, Lutz A, Davidson RJ. Reconstructing and deconstructing the self: cognitive mechanisms in meditation practice. Trends in cognitive sciences. 2015 Sep 1;19(9):515-23.

8. Could you please include (either in methods section or as supplementary material) the information that was given to participants during recruitment, including the wording of the aims of the study. Could there have been a likelihood that participants believed that the study might aim to demonstrate that meditation retreats (or vacation with meditation) are superior to vacations? If so, please discuss this as a limitation.

9. Please specify the types of meditation practices that participants in the vacation-with-meditation group engaged in. If those differed from the types of meditation practised on retreat, then please highlight and discuss this when interpreting the results.

Results

10. If available, could you please add to Table 1 the number (%) of participants in each group who have previously been on retreat.

Discussion

11. To nuance the discussion, please briefly mention potential downsides of meditation retreats that are unlikely to be encountered on vacation without intensive meditation practice. For instance, a recent meta-analysis highlights potential meditation-related challenges (Farias et al., 2020) and a large study of regular meditators indicated that individuals with retreat experience were more likely to report unpleasant meditation-related experiences (Schlosser et al., 2019).

References:

Farias M, Maraldi E, Wallenkampf KC, Lucchetti G. Adverse events in meditation practices and meditation‐based therapies: a systematic review. Acta Psychiatrica Scandinavica. 2020 Aug 7.

Schlosser M, Sparby T, Vörös S, Jones R, Marchant NL. Unpleasant meditation-related experiences in regular meditators: Prevalence, predictors, and conceptual considerations. PloS one. 2019 May 9;14(5):e0216643.

Tables

12. Please add full names of MeR, VvM, VoM, AWA etc. to the footnote of each table.
