# Supplementary material for: Is a meditation retreat the better vacation? effect of retreats and vacations on fatigue, emotional well-being, and acting with awareness
Source: PLoS One. 2021 Feb 8;16(2):e0246038. doi: 10.1371/journal.pone.0246038 (PMC7869997; doi:10.1371/journal.pone.0246038)
Supplement: Attachment — Submitted filename: Responses to reviewers revision 2.docx [file pone.0246038.s005.docx]

# **Responses to reviewers**

We would like to thank the reviewer and the editor for the renewed review of our manuscript and for the positive assessment. Below, we address the remaining issues raised by Reviewer 2 point by point.

We are looking forward to receiving your feedback on these changes.

# Reviewer #2

Points:

1. Line 113: “Thus, within a retreat, meditation practice invites a practitioner to be more open and non-judgmental to one’s experience, without labeling them as pleasing or unpleasing, through repetitive familiarization with one’s cognitive processes as well as the content of one’s consciousness. As such, meditation is geared at “seeing things as they are “.
   We deleted this section as suggested. We understand that this statement may have been unsatisfactory in various ways and did not reflect the diversity of meditation practices sufficiently.
2. Line 147: corrected
3. Line 168: “… by various psychological mechanisms associated with an increase in mindfulness.” We changed “associated with” to “including” as suggested.
4. Discussion, end of 4^th^ paragraph: We thank the reviewer for this suggestion and added the following paragraph: *“It should be noted that other psychological processes associated with meditation practice not included in this study might also play a role in improving well-being, such as self-compassion [60,61], neuroticism, and perceived control [31]. We suggest that future research on vacation, meditation retreats, and regular meditation practice should consider a broader range of potential mediators.”* We have also included the suggested literature (Baer, 2012; Schlosser, 2020).
5. Following the advice to add one sentence regarding the relatively short well-being measures we used, we added the following paragraph in the discussion: *“Fourthly, we used a relatively short scale (i.e., 5 items) to assess emotional well-being. Even though this scale has good psychometric qualities demonstrating high levels of reliability, validity as well as sensitivity [66], future research could replicate our findings with more comprehensive scales and broaden the well-being construct to include eudaimonic well-being, thriving and meaning [67,68].”* In addition, the results for emotional well-being and fatigue are comparable in our study, thus supporting the validity of the WHO-5 measure.
